# Pan-Immune-Inflammation Index as a Biomarker for Rheumatoid Arthritis Progression and Diagnosis

**DOI:** 10.7759/cureus.46609

**Published:** 2023-10-06

**Authors:** Duygu Tutan, Ayşe G Doğan

**Affiliations:** 1 Department of Internal Medicine, Erol Olçok Research and Training Hospital, Çorum, TUR; 2 Department of Physical Medicine and Rehabilitation, Erol Olçok Research and Training Hospital, Çorum, TUR

**Keywords:** rheumatology, disease activity, internal medicine, pan-immune-inflammation value, rheumatoid arthritis

## Abstract

Introduction

Rheumatoid arthritis (RA) is a chronic autoimmune condition that causes systemic inflammation and affects multiple joints. It is characterized by joint warmth, swelling, pain, and the formation of invasive synovial tissue known as pannus, which contributes to cartilage and bone degradation. Pan-immune-inflammation value (PIV), a marker derived from complete blood count parameters, has shown promise in predicting prognosis in various cancer types and pediatric conditions associated with immune abnormalities. This study aims to explore the relationship between RA, characterized by chronic inflammation and immune system involvement, and PIV, potentially shedding light on novel insights into RA's clinical implications.

Methods

One hundred four participants, including 64 RA patients (both newly diagnosed and established cases) and 40 healthy controls, were included in the study. Exclusion criteria for RA patients included acute infection, cancer, diabetes, or chronic illness, while control participants were excluded for inflammatory disorders, active infection, diabetes, or malignancy. We assessed disease severity using Disease Activity Score 28 (DAS 28) and obtained complete blood count values, including neutrophil, lymphocyte, platelet, monocyte, and red cell distribution width. C-reactive peptide (CRP) and erythrocyte sedimentation rate were also added. Statistical analyses included correlation assessments, t-tests, Mann-Whitney U tests, and multivariate linear regression. A multiclass receiver operating characteristic analysis determined optimal PIV cut-off values for distinguishing control, remission, and active RA groups, with sensitivity, specificity, positive predictive value (PPV), negative predictive value (NPV), accuracy, and odds ratios calculated.

Results

This study comprised a cohort of 104 participants, with a median age of 43.5±17.5. The Remission group was significantly younger than the Control group (p=0.006) but not compared to the Active RA group (p=0.393). CRP levels were significantly higher in the Active RA group (p<0.001). Neutrophil counts were highest in the Active RA group (p<0.001), as were monocyte counts. Lymphocyte counts were significantly lower in the Active RA group (p<0.001). There were no significant differences in sedimentation rate, hemoglobin, platelet count, and mean platelet volume. PIV was significantly elevated in the Active RA group (p<0.001) and higher in the Remission group than in the Control group (p=0.001). A PIV value of 353.48 exhibited 71.4% sensitivity, 86.2% specificity, 86.2% PPV, 71.4% NPV, and 78.13% test accuracy for distinguishing active rheumatoid arthritis (p<0.001). A PIV value exceeding 353.48 substantially increased the likelihood of a patient belonging to the active rheumatoid arthritis group, with a 14.62-fold higher probability. Furthermore, the study explored the relationship between clinical and laboratory variables and disease activity in RA patients, finding significant differences in PIV among DAS groups (p=0.025).

Conclusions

The PIV offers a notable advantage as its constituent parameters are routinely assessed in rheumatoid arthritis and involve cost-effective and straightforward tests. We demonstrated that PIV serves as a valuable marker for distinguishing between remission and active RA when compared to healthy individuals. Additionally, it proved to be an effective tool for assessing disease activity in patients with active rheumatoid arthritis.

## Introduction

Rheumatoid arthritis (RA) is a chronic autoimmune condition characterized by systemic inflammation that affects numerous joints [1]. Key pathological elements include joint warmth, swelling, and pain. Concurrently, pannus, a type of invasive synovial tissue, emerges, contributing to the degradation of cartilage and bone. These cardinal features collectively define the complex landscape of RA. Moreover, RA's impact is not confined solely to joint involvement, encompassing diverse systemic manifestations such as dermatological symptoms, fever, muscle wasting, and weakness [2-5]. Rheumatoid arthritis exhibits a higher prevalence among women, particularly in their 40s [6]. Earlier investigations have underscored the intricate nature of RA's pathogenesis. This complexity is primarily demonstrated at the cellular level, characterized by a dysregulation in the equilibrium between osteoblasts and osteoclasts, aberrant synoviocyte proliferation, and malfunction of immune cells [7].

Cellular inflammatory mediators, including cytokines such as interleukin (IL)-17, tumor necrosis factor-alpha (TNF-α), IL-6, and IL-8, can contribute to the development of rheumatoid arthritis [1]. Contemporary investigations have shed light on the pivotal role of autoreactive T cells in the pathological progression of RA [8]. Naïve CD4 T cells undergo differentiation into pro-inflammatory helper T cells, characterized by an enhanced capacity to infiltrate tissues and incite inflammation via immune cell-mediated cytotoxicity [8,9].

The pan-immune-inflammation value (PIV) is a marker that is calculated from the complete blood count and is used to assess the severity of inflammation. It is derived from four blood cell counts, including neutrophils, platelets, monocytes, and lymphocytes. The PIIV has been validated as a predictor of prognosis in various oncological diseases [10]. It has been shown to be associated with clinical outcomes and tumor-infiltrating lymphocytes in esophageal cancer [11]. Additionally, the PIV has been reported as a promising predictor of long-term outcomes in colorectal cancer patients [12].

The PIV has also been studied in the context of breast cancer. It has been found to have prognostic potential in breast cancer patients treated with neoadjuvant chemotherapy [13]. Furthermore, a study investigated the relationship between the PIV and overall survival in patients with operative breast cancer and found that the PIV was a new prognostic index [14].

PIV has been proposed as a biomarker in other medical conditions as well. It has been evaluated in the diagnosis of respiratory distress syndrome in preterm infants [15]. In the field of pediatric acute-onset neuropsychiatric syndrome (PANS), which is associated with immune abnormalities, PIV has been studied in the context of treatment outcomes [16]. In this study, we aimed to investigate the relationship between rheumatoid arthritis, a disease affecting the immune system and characterized by chronic inflammation, and PIV.

## Materials and methods

This investigation received authorization from the Hitit University Clinical Research Ethics Committee (September 13, 2023, approval 2023-116), and all protocols undertaken in investigations involving human subjects were conducted in strict compliance with the ethical guidelines outlined by the institutional and/or national research governing body, the 1964 Declaration of Helsinki, and its subsequent revisions or analogous ethical criteria. The research cohort comprised 104 individuals, encompassing 64 individuals diagnosed with RA, either as newly identified cases or previously established patients, along with a control group consisting of 40 healthy individuals. These participants were subjected to examination within the Departments of Physical Therapy and Rehabilitation and Internal Medicine at Hitit University's Erol Olçok Training and Research Hospital during the period spanning September 1, 2019, to August 20, 2023. The exclusion criteria for the patient group were acute infection, cancer, diabetes, or usage of drugs that can affect neutrophil and lymphocyte levels, such as corticosteroids or granulocyte stimulating factors, whereas the exclusion criteria for the control group were inflammatory disorders, chronic or acute infection, diabetes, active malignancy and usage drugs that can affect neutrophil and lymphocyte levels, such as corticosteroids or granulocyte stimulating factors. Disease Activity Score 28 (DAS 28), the complete blood count values of the patient group, and the complete blood count values of the control group were obtained from the patient information system. A DAS 28 exceeding 5.1 is indicative of a high level of disease activity (classified as level 3), while a DAS 28 range of 5.1 to 3.2 signifies moderate disease activity (level 2), and a range of 2.6 to 3.2 represents low disease activity (level 1). Values falling below 2.6 are considered to reflect a state of remission.

The study included neutrophil, lymphocyte, platelet, monocyte, and red cell distribution width (RDW) values derived from routine hematological analyses within both the patient and control cohorts. Subsequently, a comparative analysis was conducted to assess immune-inflammatory parameters between these two groups. Furthermore, these parameters were subjected to a stratified comparison based on the DAS 28 activity levels within the patient cohort.

This study was retrospectively designed. Statistical analysis was conducted utilizing IBM SPSS Statistics for Windows software, version 26, developed by IBM Corp., Armonk, NY, USA. Descriptive statistics were presented in the form of counts and percentages for categorical variables, the mean ± standard deviation (SD) for variables demonstrating a normal distribution, and the median ± interquartile range (IQR) for variables exhibiting non-normally distributed patterns. The assessment of data distribution was performed using the Shapiro-Wilk test. Accordingly, Pearson and Spearman correlation coefficients were employed to scrutinize the associations between the various variables, depending on the distribution characteristics of the data.

Comparative analysis of numerical variables between groups was executed through the utilization of the Student's t-test and the Mann-Whitney U test, contingent upon the data's distribution characteristics. Meanwhile, comparisons of categorical variable ratios among research groups were assessed via the Chi-square test. Subsequently, a multivariate linear regression analysis was employed to ascertain the relationship between the PIV and DAS 28 severity scores, with adjustments made for confounding factors.

To distinguish between the control, remission, and active rheumatoid arthritis groups, a multiclass receiver operating characteristic (ROC) analysis was conducted. Optimal PIV cut-off values were determined using the area under the curve (AUC) and the Youden index. Corresponding to these cut-off values, sensitivity, specificity, positive predictive value (PPV), negative predictive value (NPV), test accuracy, and odds ratio values were computed. A significance level of p < 0.05 was deemed statistically significant.

## Results

This study encompassed a cohort of 104 individuals, comprising 58.65% males and 41.35% females, with a median age of 43.5±17.5. Among them, 64 participants (61.54%) received a diagnosis of rheumatoid arthritis. Comprehensive clinical attributes of all subjects are presented in Table 1.

**Table 1:** Descriptive characteristics of all participants and comparison results of subgroups RA: rheumatoid arthritis, CRP: C-reactive peptide, MPV: mean platelet volume, RDW: red cell distribution width, PIV: pan-immune-inflammatory value, DAS 28: Disease Activity Score 28

Variables		All Participants	Control	Remission	Active RA	Statistical Significance	Control vs Remission	Control vs Active RA	Remission vs Active RA
Age (years)		43.5±17.5	48±15	36±11	41±13	0.015	0.006	0.046	0.393
Gender	Male	61 (58.65%)	22 (55%)	14 (48.28%)	25 (71.43%)	0.145			
	Female	43 (41.35%)	18 (45%)	15 (51.72%)	10 (28.57%)				
CRP (mg/dL)		3.95±7.12	3.22±1.92	3.11±1	12.8±14	<0.001	0.143	<0.001	<0.001
Sedimentation Rate		8±14	8±12.5	8±9	11±17	0.278			
Hemoglobin (mg/dL)		14±2.6	14.95±2.5	13.8±3	13.9±2.5	0.069			
Neutrophil (10^9^/L)		4.78±2	4.08±1.66	4.18±1.21	5.76±2.08	<0.001	0.968	<0.001	<0.001
Monocyte (10^9^/L)		0.53±0.16	0.41±0.2	0.57±0.06	0.57±0.11	<0.001	<0.001	<0.001	0.821
Lymphocyte (10^9^/L)		2.28±0.8	2.42±0.55	2.3±0.68	1.85±0.89	<0.001	0.060	<0.001	0.021
Platelet (10^9^/L)		254.5±75.5	252.5±83	268±72	252±79	0.741			
MPV		10.1±1.2	10.2±1.15	10.1±1.2	10.06±1.2	0.671			
RDW		13.7±1.3	13.15±1.2	13.8±1.2	14.2±1.5	0.002	0.004	0.002	0.948
PIV		245.1±296.22	149.64±108.57	240.35±113.96	471.61±365.52	<0.001	0.001	<0.001	0.003
DAS 28 Score				1.74±1.03	4.87±2.17				

Comparison of control, remission, and active rheumatoid arthritis groups

Notably, individuals in the Remission group displayed a statistically significant younger mean age (36 years ± 11) compared to the Control group (48 years ± 15) but there was no significance observed when compared to the Active RA group (41 years ± 13) (p = 0.006 and p = 0.393, respectively). Gender distribution revealed variations among groups, but these differences were not statistically significant (p = 0.145). The C-reactive protein (CRP) levels exhibited significant differences among groups. Participants in the Active RA group had markedly elevated CRP levels (12.8 mg/dL ± 14) compared to the Control group (3.22 mg/dL ± 1.92) and the Remission group (3.11 mg/dL ± 1) (p < 0.001 for both comparisons).

**Figure 1:**
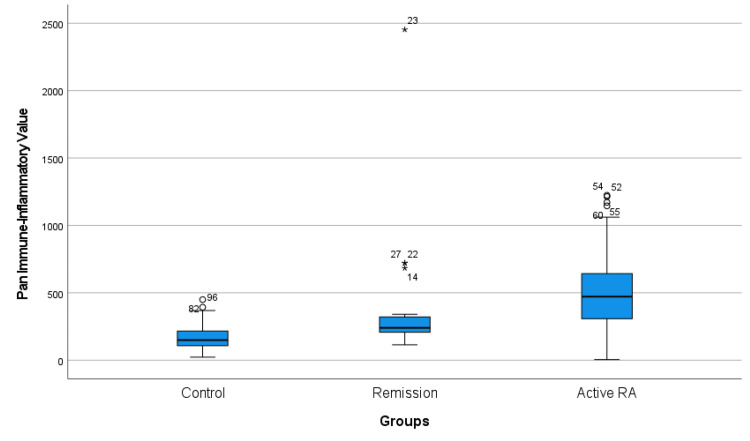
Boxplot diagram of pan-immune-inflammatory index of study groups o: Mild outlier, *: Extreme outlier RA: rheumatoid arthritis

Neutrophil counts also displayed significant variability, with the Active RA group showing the highest mean (5.76 ± 2.08) compared to the Control group (4.08 ± 1.66) and the Remission group (4.18 ± 1.21) (p < 0.001 for both comparisons). Similar significant differences were observed in monocyte count among the groups. The Active RA group displayed a statistically significant reduction in lymphocyte count, with a mean of 1.85 ± 0.89, compared to both the Control group (2.42 ± 0.55) and the Remission group (2.3 ± 0.68) (p < 0.001). There were no significant differences between groups in terms of sedimentation rate, hemoglobin, platelet count, and mean platelet volume (MPV) (p = 0.278, p = 0.069, p = 0.741, p = 0.671, respectively) (Table 1). Our primary variable of interest, PIV, demonstrated substantial disparities among the study groups. PIV levels in the Active RA group were strikingly elevated (471.61 ± 365.52) in comparison to the Control group (149.64 ± 108.57) and the Remission group (240.35 ± 113.96) (p < 0.001 for both comparisons). This pronounced elevation in PIV levels underscores the heightened immune-inflammatory state in individuals with active RA. PIV was also higher in the Remission group than the Control group (p = 0.001) (Figure 1).

**Figure 2:**
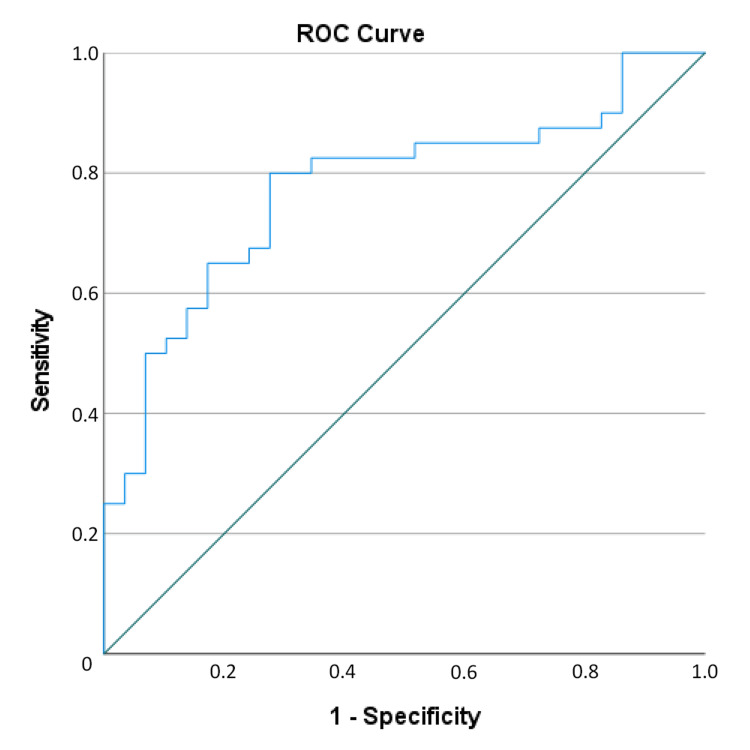
Receiver operating curve (ROC) of distinction between Control and Remission groups

**Figure 3:**
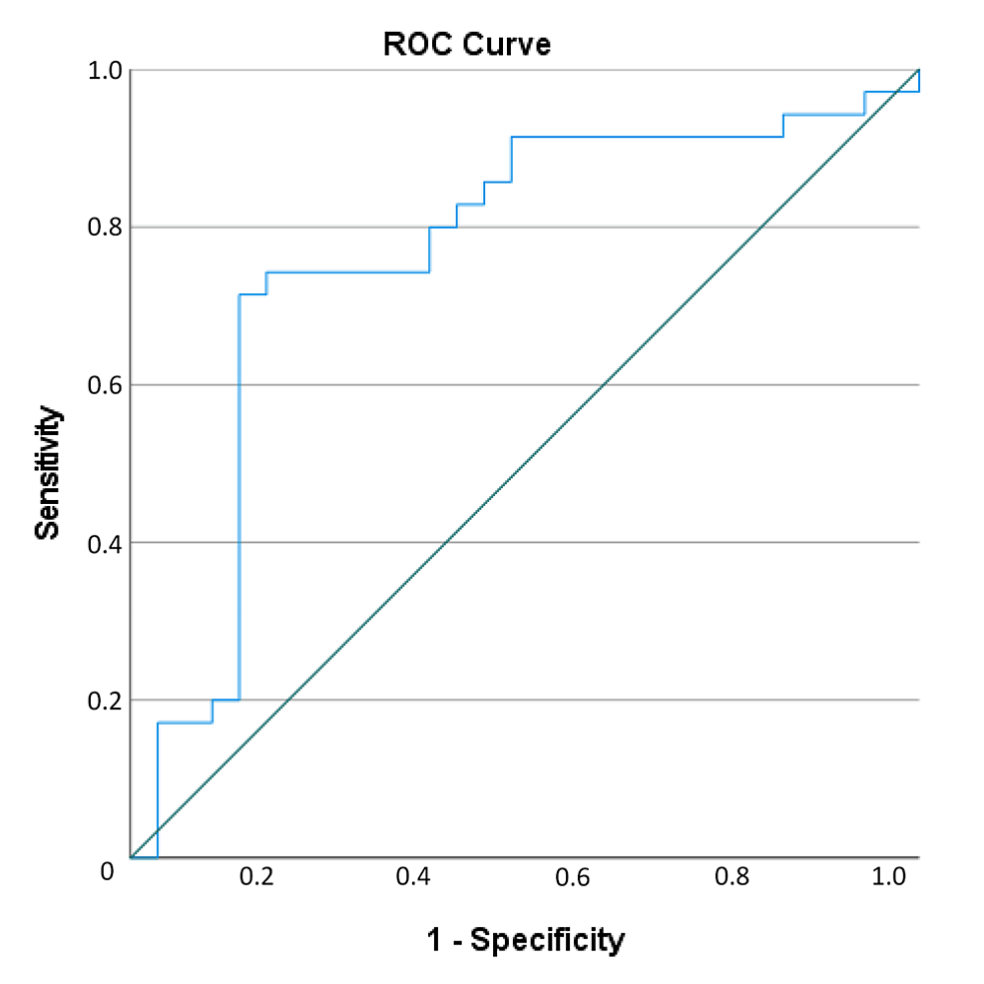
Receiver operating curve (ROC) of the distinction between Active Rheumatoid Arthritis and Remission groups

In the ROC analysis performed to find the optimal values of PIV for distinction between control, remission and active RA groups, a value of 217.53 was found optimal for PIV with 72.4% sensitivity, 80.0% specificity, 72.4% PPV, 80.0% NPV and 76.81% test accuracy for the distinction between control and remission patients, and a PIV value of 353.48 was found with 71.4% sensitivity, 86.2% specificity, 86.2% PPV, 71.4% NPV and 78.13% test accuracy for the distinction of active rheumatoid arthritis (Figures 2, 3). A PIV of 353.48 and above increased the likelihood of the patient being in the active rheumatoid arthritis group 14.62-fold (Table 2).

**Table 2:** Diagnostic value of pan-immune-inflammatory value cut-offs PIV: pan-immune-inflammation value, rem: remission group, Contr: control group, RA: rheumatoid arthritis, PPV: positive predictive value, NPV: negative predictive value, ROC: receiver operating curve, SE: standard error, CI: confidence interval OR: odds ratio

Variables	Cut-Off	Diagnostic Values					ROC Analysis			Odds Ratio		
		Sensitivity	Specificity	PPV	NPV	Accuracy	Area (SE)	%95 CI	p	OR	%95 CI	p
PIV (Contr. vs Rem.)	217.53	72.4	80.0	72.4	80.0	76.81	0.775 (0.057)	0.664-0.886	<0.001	10.500	3.412-32.309	<0.001
PIV (Rem vs Active RA)	353.48	71.4	86.2	86.2	71.4	78.13	0.763 (0.065)	0.636-0.889	<0.001	15.625	4.322-56.492	<0.001

Comparison of variables according to disease activity level in rheumatoid arthritis patients

To investigate the association between various clinical and laboratory variables and disease activity in patients with rheumatoid arthritis, a comprehensive analysis was performed between groups divided according to the Disease Activity Score. No statistically significant difference was observed between the three groups regarding demographic characteristics (p = 0.785 for age, p = 0.154 for gender).

In the context of inflammatory markers, it was observed that CRP levels exhibited statistically significant differences across the three Disease Activity Score categories (p = 0.001). Subsequent post-hoc analysis unveiled notable distinctions in CRP levels, specifically between DAS I and DAS III (p = 0.003) and between DAS II and DAS III (p = 0.001). Conversely, the sedimentation rate demonstrated no statistically significant disparity among the three DAS groups (p = 0.075). Regarding hematological parameters, hemoglobin, platelet count, MPV, and RDW levels did not display significant variations between DAS groups (p = 0.853, p = 0.385, p = 0.109, and p = 0.623, respectively). In contrast, neutrophil, monocyte, and lymphocyte counts exhibited a statistically significant discrepancy among the three groups (p = 0.012, p = 0.030, p = 0.002) (Table 3).

**Table 3:** Descriptive characteristics of all participants and comparison results of subgroups RA: rheumatoid arthritis. CRP: C-reactive peptide. MPV: mean platelet volume. RDW: red cell distribution width. PIV: pan-immune-inflammatory value. DAS 28: Disease Activity Score 28

Variables		DAS I	DAS II	DAS III	Statistical Significance	DAS I vs DAS II	DAS I vs DAS III	DAS II vs DAS III
Age (years)		45±8	39.5±11	42.5±15	0.785			
Gender	Male	3 (60%)	8 (57.14%)	14 (87.5%)	0.154			
	Female	2 (40%)	6 (42.86%)	2 (12.5%)				
CRP (mg/dL)		4.98±2.19	11±9.04	19.15±21	0.001	0.221	0.001	0.003
Sedimentation Rate		6±7	7±16	19.5±21	0.075			
Hemoglobin (mg/dL)		13.8±0.9	13.8±3	13.9±2.7	0.853			
Neutrophil (10^9^/L)		5.24±0.57	5.6±0.85	7.16±2.34	0.012	0.547	0.017	0.013
Monocyte (10^9^/L)		0.75±0.2	0.56±0.07	0.51±0.19	0.030	0.032	0.008	0.521
Lymphocyte (10^9^/L)		2.65±0.5	1.89±0.53	1.47±0.61	0.002	0.057	0.001	0.042
Platelet (10^9^/L)		263±13	243±52	247±84.5	0.385			
MPV		9.5±1.7	10.45±0.9	9.8±1.15	0.109			
RDW		13.3±0.5	14.3±1.2	14±2.55	0.623			
PIV		471.61±102.49	435.7±206.06	642.82±719.59	0.025	0.856	0.097	0.010
DAS 28 Score		3.1±0.08	4.52±0.89	6.14±0.81	<0.001			

**Figure 4:**
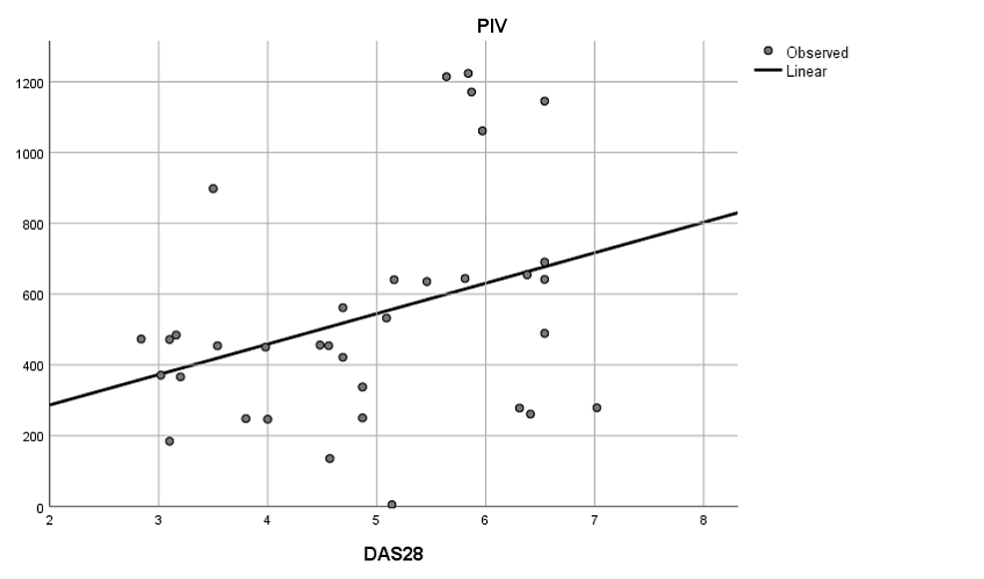
Linear graph of the relation between pan-immune-inflammatory value (PIV) and Disease Activity Score 28 (DAS 28)

The PIV displayed a statistically significant difference between DAS I, DAS II, and DAS III (p = 0.025). Subsequent post-hoc analysis elucidated significant differences in PIV, particularly between DAS I and DAS III (p = 0.010) (Table 3, Figure 4).

## Discussion

RA is a complex and systemic disease leading to joint and periarticular destruction. Cellular and humoral immunity play a role together in the pathogenesis of the disease [17]. Neutrophils are the precursor cells of the immune system, and these cells synthesize cytokines, chemokines, and growth factors. Platelets also contribute to the increase in cytokines that occur in inflammation. In addition, they stimulate neutrophil and platelet synthesis and contribute to the continuation of inflammation [18,19]. In inflammatory events, an increase in the number of neutrophils, platelets, and monocytes and a decrease in the number of lymphocytes are observed [19]. In our study, when the rheumatoid arthritis group was compared with the control group, the number of neutrophils and monocytes in the patient group and the number of lymphocytes in the control group were found to be higher. When the patient group was compared according to the DAS level, neutrophil and monocyte levels were found to be significantly higher with increasing inflammation, while lymphocyte levels were found to be significantly lower in accordance with the previous literature.

C-reactive protein is an acute-phase reactant produced by the liver in response to inflammation. It is commonly used as a marker of inflammation and disease activity in various inflammatory conditions, including RA [20]. Another study suggested that CRP levels directly correlate with the disease activity of inflammatory diseases [21]. The substantial variations in CRP levels across disease activity categories in our study are consistent with the well-established role of CRP as a sensitive indicator of systemic inflammation in RA. The markedly elevated CRP levels in active RA patients corroborate its utility in distinguishing disease states. Importantly, post-hoc analysis further highlights CRP's clinical relevance in differentiating between control, remission, and active RA groups, reinforcing its role as a valuable biomarker.

The erythrocyte sedimentation rate (ESR) is a commonly used laboratory test to assess inflammation and disease activity in various conditions, including rheumatoid arthritis [22]. Several studies have investigated the relationship between CRP, ESR, and RA and reported different sensitivity rates [22,23]. The lack of statistically significant differences in sedimentation rate among disease activity groups reaffirms the notion that sedimentation rate may possess lower sensitivity than CRP in reflecting RA disease activity. This finding underscores the importance of considering multiple markers in disease assessment to capture the full spectrum of inflammatory processes in RA.

PIV is a marker that integrates various markers of inflammation and immune system and has shown promise as a predictor of prognosis in different diseases [15,16]. It has been studied in various cancer types, including esophageal, colorectal, and breast cancer [11-13]. PIV's ability to capture the inflammatory changes in immune and inflammatory parameters commonly seen in rheumatoid arthritis makes it a potentially useful tool for assessing the level of systemic inflammation in RA. In our study, PIV also displayed significant differences between DAS I, DAS II, and DAS III, showing its potential as a composite biomarker reflecting overall immune-inflammatory status in RA patients.

## Conclusions

In conclusion, the main advantage of the PIV is that the parameters that constitute PIV are routinely assessed in rheumatoid arthritis and are inexpensive and simple tests. According to our findings in this study, the pan-immune-inflammatory value was shown to be a useful marker for assessing remission and active rheumatoid arthritis in contrast to healthy participants, as well as disease activity in active rheumatoid arthritis patients.
